# Correction: Genome size influences plant growth and biodiversity responses to nutrient fertilization in diverse grassland communities

**DOI:** 10.1371/journal.pbio.3003195

**Published:** 2025-05-20

**Authors:** Joseph A. Morton, Carlos Alberto Arnillas, Lori Biedermann, Elizabeth T. Borer, Lars A. Brudvig, Yvonne M. Buckley, Marc W. Cadotte, Kendi Davies, Ian Donohue, Anne Ebeling, Nico Eisenhauer, Catalina Estrada, Sylvia Haider, Yann Hautier, Anke Jentsch, Holly Martinson, Rebecca L. McCulley, Xavier Raynaud, Christiane Roscher, Eric W. Seabloom, Carly J. Stevens, Katerina Vesela, Alison Wallace, Ilia J. Leitch, Andrew R. Leitch, Erika I. Hersch-Green

There are errors in the Author Contributions. The correct contributions are:

**Conceptualization:** Joseph A. Morton, Carlos Alberto Arnillas, Ilia J. Leitch, Andrew R. Leitch, Erika I. Hersch-Green.

**Data curation:** Joseph A. Morton, Carlos Alberto Arnillas, Lori Biedermann, Elizabeth T. Borer, Lars A. Brudvig, Yvonne M. Buckley, Marc W. Cadotte, Kendi Davies, Ian Donohue, Anne Ebeling, Nico Eisenhauer, Catalina Estrada, Sylvia Haider, Yann Hautier, Anke Jentsch, Holly Martinson, Rebecca L. McCulley, Xavier Raynaud, Christiane Roscher, Eric W. Seabloom, Carly J. Stevens, Katerina Vesela, Alison Wallace, Ilia J. Leitch, Erika I. Hersch-Green.

**Formal analysis:** Joseph A. Morton, Marc W. Cadotte, Yann Hautier, Erika I. Hersch-Green.

**Funding acquisition:** Elizabeth T. Borer, Erika I. Hersch-Green.

**Investigation:** Joseph A. Morton, Ilia J. Leitch, Andrew R. Leitch, Erika I. Hersch-Green.

**Methodology:** Joseph A. Morton, Carlos Alberto Arnillas, Lori Biedermann, Lars A. Brudvig, Yvonne M. Buckley, Marc W. Cadotte, Kendi Davies, Ian Donohue, Anne Ebeling, Nico Eisenhauer, Catalina Estrada, Sylvia Haider, Yann Hautier, Anke Jentsch, Holly Martinson, Rebecca L. McCulley, Christiane Roscher, Eric W. Seabloom, Carly J. Stevens, Katerina Vesela, Alison Wallace, Erika I. Hersch-Green.

**Project administration:** Joseph A. Morton, Elizabeth T. Borer, Ilia J. Leitch, Andrew R. Leitch, Erika I. Hersch-Green.

**Resources:** Lori Biedermann, Elizabeth T. Borer, Xavier Raynaud, Erika I. Hersch-Green.

**Supervision:** Joseph A. Morton, Ilia J. Leitch, Andrew R. Leitch, Erika I. Hersch-Green.

**Validation:** Joseph A. Morton, Erika I. Hersch-Green.

**Visualization** Joseph A. Morton, Erika I. Hersch-Green.

**Writing – original draft:** Joseph A. Morton, Erika I. Hersch-Green.

**Writing – review & editing:** Joseph A. Morton, Carlos Alberto Arnillas, Lori Biedermann, Elizabeth T. Borer, Lars A. Brudvig, Yvonne M. Buckley, Marc W. Cadotte, Kendi Davies, Ian Donohue, Anne Ebeling, Nico Eisenhauer, Catalina Estrada, Sylvia Haider, Yann Hautier, Anke Jentsch, Holly Martinson, Rebecca L. McCulley, Xavier Raynaud, Christiane Roscher, Eric W. Seabloom, Carly J. Stevens, Katerina Vesela, Alison Wallace, Ilia J. Leitch, Andrew R. Leitch, Erika I. Hersch-Green.

The following information is missing from the Acknowledgement section: The authors thank the Nutrient Network (http://www.nutnet.org) for providing long-term grassland site data, the Minnesota Supercomputer Institute for hosting Nutrient Network project data, and the Institute on the Environment for hosting Network meetings. They also thank Angela Walczyk and Hailee Hass for providing flow cytometry and cell count data. This research is KBS Contribution #2,399.
