# Supplementary material for: Genome size influences plant growth and biodiversity responses to nutrient fertilization in diverse grassland communities
Source: PLoS Biol. 2024 Dec 11;22(12):e3002927. doi: 10.1371/journal.pbio.3002927 (PMC11633961; doi:10.1371/journal.pbio.3002927)
Supplement: S1 Table — Name, location, and study period of each site is given, as well as habitat type, elevation, climate, number of replication blocks, and the proportion of species with available genome size data (GS coverage). The climate variables used are taken from WorldClim v.2 30s data: Annual mean temperature (MAT, BIO1), mean annual precipitation (MAP, BIO12), Temperature Seasonality (BIO4), and Precipitation Seasonality (BIO15). (DOCX) [file pbio.3002927.s001.docx]

**S1 Table: List of Nutrient Network sites used in this study**
