# Supplementary material for: Genome size influences plant growth and biodiversity responses to nutrient fertilization in diverse grassland communities
Source: PLoS Biol. 2024 Dec 11;22(12):e3002927. doi: 10.1371/journal.pbio.3002927 (PMC11633961; doi:10.1371/journal.pbio.3002927)
Supplement: S2 Table — Results of linear mixed-effects models fitting the effect of N and P fertilization on the change in cover-weighted GS, compared to control plots (ΔcwGS(control vs. treatment, a, b) and compared to pretreatment levels (ΔcwGS(pretreatment vs. treatment), c, d) (n = 681). In addition, the summary table is presented for a model fitting the effects of nutrients, temperature, and precipitation on the log response ratio (LRR) of cover-weighted GS (ΔcwGS(control vs. treatment)). (e) Models in a–d were also repeated including only the species for which directly measured GS values exist, only on sites where they were sampled. (f–i) Significant differences are shown in bold and starred (* = p ≤ 0.05, ** = p ≤ 0.01, *** = p ≤ 0.001). (DOCX) [file pbio.3002927.s002.docx]

**S2 Table ANOVA outputs of weighted genome size (GS) models**

**a** – ANOVA table for the effect of N and P fertilisation on ΔcwGS_(control vs treated)_. R^2^ = 0.069.

| **ΔcwGS (control vs treated) ~** | **Sum Sq** | **Mean Sq** | **df** | **F-value** | **p-value** |  |
| --- | --- | --- | --- | --- | --- | --- |
| **N added** | **1.72** | **1.72** | **1, 592** | **18.96** | **<0.001** | ******* |
| P added | 0.11 | 0.11 | 1, 592 | 1.20 | 0.274 |  |
| N added : P added | 0.11 | 0.11 | 1, 591 | 1.21 | 0.271 |  |

**b** – Summary table for effect of N and P fertilisation on ΔcwGS_(control vs treated)_.

| **ΔcwGS (control vs treated) ~** | **Estimate** | **Standard Error** | **df** | **t-value** | **p-value** |  |
| --- | --- | --- | --- | --- | --- | --- |
| No nutrients added | -0.050 | 0.032 | 59 | -1.55 | 0.127 |  |
| **N added** | **0.142** | **0.033** | **592** | **4.35** | **<0.001** | ******* |
| P added | 0.036 | 0.033 | 592 | 1.10 | 0.274 |  |
| N and P added | 0.051 | 0.046 | 591 | 1.10 | 0.271 |  |

**c** – ANOVA table for the effect of N and P fertilisation on ΔcwGS_(pretreatment vs treatment)_. R^2^ = 0.053.

| **ΔcwGS (pretreatment vs treatment) ~** | **Sum Sq** | **Mean Sq** | **df** | **F-value** | **p-value** |  |
| --- | --- | --- | --- | --- | --- | --- |
| **N added** | **2.08** | **2.08** | **1, 589** | **18.46** | **<0.001** | ******* |
| P added | 0.26 | 0.26 | 1, 589 | 2.33 | 0.128 |  |
| N added : P added | 0.06 | 0.06 | 1, 588 | 0.57 | 0.451 |  |

**d** – Summary table for effect of N and P fertilisation on ΔcwGS_(pretreatment vs treatment)._

| **ΔcwGS (pretreatment vs treatment) ~** | **Estimate** | **Standard Error** | **df** | **t-value** | **p-value** |  |
| --- | --- | --- | --- | --- | --- | --- |
| No nutrients added | -0.071 | 0.050 | 37 | -1.41 | 0.166 |  |
| **N added** | **0.156** | **0.036** | **589** | **4.30** | **<0.001** | ******* |
| P added | 0.055 | 0.036 | 589 | 1.53 | 0.128 |  |
| N and P added | 0.039 | 0.052 | 588 | 0.75 | 0.451 |  |

**e** – Summary table for effect of N and P fertilisation and four climatic variables on ΔcwGS_(control vs treated)._

| **Log response ratio of weighted genome size ~** | **Estimate** | **Standard Error** | **df** | **t-value** | **p-value** |  |
| --- | --- | --- | --- | --- | --- | --- |
| No nutrients added | -0.053 | 0.030 | 55 | -1.76 | 0.084 |  |
| **N added** | **0.143** | **0.032** | **579** | **4.45** | **<0.001** | *** |
| P added | 0.036 | 0.032 | 579 | 1.12 | 0.263 |  |
| N added : P added | 0.051 | 0.046 | 579 | 1.11 | 0.268 |  |
| Mean Temperature | -0.002 | 0.032 | 55 | -0.08 | 0.939 |  |
| Mean Precipitation | -0.006 | 0.033 | 69 | -0.18 | 0.854 |  |
| Temperature Seasonality | 0.016 | 0.035 | 58 | 0.45 | 0.651 |  |
| Precipitation Seasonality | -0.008 | 0.036 | 59 | -0.22 | 0.825 |  |
| N : Temperature | 0.009 | 0.034 | 578 | 0.27 | 0.790 |  |
| P : Temperature | -0.006 | 0.034 | 578 | -0.19 | 0.851 |  |
| N and P : Temperature | -0.025 | 0.048 | 580 | -0.53 | 0.599 |  |
| **N : Precipitation** | **-0.106** | **0.036** | **583** | **-2.98** | **0.003** | ****** |
| P : Precipitation | -0.021 | 0.036 | 583 | -0.58 | 0.564 |  |
| N and P : Precipitation | 0.004 | 0.051 | 580 | 0.07 | 0.941 |  |
| **N : Temp. Seasonality** | **-0.075** | **0.037** | **577** | **-2.06** | **0.040** | ***** |
| P : Temp. Seasonality | -0.069 | 0.037 | 577 | -1.88 | 0.061 |  |
| N and P : Temp. Seasonality | 0.096 | 0.052 | 577 | 1.86 | 0.064 |  |
| N : Precip. Seasonality | -0.006 | 0.039 | 583 | -0.15 | 0.883 |  |
| P : Precip. Seasonality | -0.005 | 0.039 | 583 | -0.14 | 0.893 |  |
| N and P : Precip. Seasonality | 0.024 | 0.055 | 581 | 0.43 | 0.670 |  |

**f** – ANOVA table for the effect of N and P fertilisation on ΔcwGS_(control vs treated)_, only including species for which directly measured GS values are available. R^2^ = 0.096.

| **ΔcwGS (control vs treated) ~** | **Sum Sq** | **Mean Sq** | **df** | **F-value** | **p-value** |  |
| --- | --- | --- | --- | --- | --- | --- |
| **N added** | **0.677** | **0.677** | **1, 336** | **5.59** | **0.019** | ***** |
| P added | 0.088 | 0.088 | 1, 336 | 0.73 | 0.395 |  |
| **N added : P added** | **1.094** | **1.094** | **1, 335** | **9.04** | **0.003** | ****** |

**g** – Summary table for effect of N and P fertilisation on ΔcwGS_(control vs treated)_, only including species for which directly measured GS values are available.

| **ΔcwGS (control vs treated) ~** | **Estimate** | **Standard Error** | **df** | **t-value** | **p-value** |  |
| --- | --- | --- | --- | --- | --- | --- |
| No nutrients added | -0.073 | 0.055 | 24 | -1.31 | 0.202 |  |
| **N added** | **0.118** | **0.050** | **336** | **2.36** | **0.019** | ***** |
| P added | -0.042 | 0.050 | 336 | -0.85 | 0.395 |  |
| **N and P added** | **0.214** | **0.071** | **335** | **3.01** | **0.003** | ** |

**h** – ANOVA table for the effect of N and P fertilisation on ΔcwGS_(pretratment vs treatment)_, only including species for which directly measured GS values are available. R^2^ = 0.058.

| **ΔcwGS (pretreatment vs treatment) ~** | **Sum Sq** | **Mean Sq** | **df** | **F-value** | **p-value** |  |
| --- | --- | --- | --- | --- | --- | --- |
| **N added** | **0.893** | **0.893** | **1, 333** | **6.26** | **0.013** | ***** |
| P added | 0.076 | 0.076 | 1, 333 | 0.53 | 0.465 |  |
| **N added : P added** | **0.790** | **0.790** | **1, 332** | **5.53** | **0.019** | * |

**i** – Summary table for effect of N and P fertilisation on ΔcwGS_(pretratment vs treatment)_, only including species for which directly measured GS values are available.

| **ΔcwGS (pretreatment vs treatment) ~** | **Estimate** | **Standard Error** | **df** | **t-value** | **p-value** |  |
| --- | --- | --- | --- | --- | --- | --- |
| No nutrients added | -0.174 | 0.095 | 14 | -1.83 | 0.088 |  |
| **N added** | **0.136** | **0.054** | **333** | **2.50** | **0.013** | ***** |
| P added | -0.040 | 0.054 | 333 | -0.73 | 0.465 |  |
| **N and P added** | **0.182** | **0.077** | **332** | **2.35** | **0.019** | * |
