# Supplementary material for: Genome size influences plant growth and biodiversity responses to nutrient fertilization in diverse grassland communities
Source: PLoS Biol. 2024 Dec 11;22(12):e3002927. doi: 10.1371/journal.pbio.3002927 (PMC11633961; doi:10.1371/journal.pbio.3002927)
Supplement: S3 Table — Phylogenetic mixed-effects models were fitted in brms [44] to examine the effect of genome size (GS) on the change in a species’ percent cover with N, P fertilization. In addition to the model outlined in Table 2, a model was fitted to include the interaction between the above factors and plant functional group (a, n = 439). Another model was also run for grass species only, with photosynthetic pathway include as an additional explanatory variable (b, n = 72). Lastly, a model was run as in Table 2 but only including species for which direct GS measurements were available (c, n = 172). Estimated intercepts and slope values showing a slope with nonzero 95% credible intervals are highlighted in bold. (DOCX) [file pbio.3002927.s003.docx]

**S3 Table Outputs of species-level phylogenetic mixed-effect model**

| **Change in % cover ~** | **Estimate** | **Standard Error** | **Credible Intervals (95%)** | **Effective Sample Size** |
| --- | --- | --- | --- | --- |
| **b -** Grass species, split by photosynthetic pathway (n = 72) R-squared = 0.189 | | | | |
| No nutrients added | -0.84 | 7.81 | -16.83, 14.92 | 13207 |
| N added | -0.54 | 0.78 | -2.08, 1.00 | 30020 |
| P added | 0.30 | 0.78 | -1.24, 1.84 | 28939 |
| N added : P added | -0.28 | 0.84 | -1.91, 1.35 | 29507 |
| C4 grasses | 0.31 | 0.97 | -1.61, 2.23 | 26832 |
| **N : C4 grasses** | **-1.97** | **0.87** | **-3.69, -0.27** | **28546** |
| P : C4 grasses | -0.41 | 0.86 | -2.11, 1.27 | 27148 |
| N : P : C4 grasses | -1.24 | 0.90 | -3.00, 0.52 | 28463 |
| log (GS) | 0.10 | 0.82 | -1.51, 1.70 | 30076 |
| **log (GS) : N** | **1.49** | **0.58** | **0.34, 2.63** | **28249** |
| log (GS) : P | 0.37 | 0.59 | -0.78, 1.52 | 29487 |
| log (GS) : N : P | 0.97 | 0.68 | -0.36, 2.31 | 29817 |
| log (GS) : C4 grasses | 0.29 | 0.93 | -1.53, 2.11 | 28166 |
| **log (GS) : N : C4 grasses** | **-2.61** | **0.85** | **-4.26, -0.93** | **27040** |
| log(GS) : P : C4 grasses | -0.20 | 0.85 | -1.87, 1.47 | 28224 |
| log (GS) : N : P : C4 grasses | -1.42 | 0.90 | -3.17, 0.34 | 27722 |
| c - Measured species (n = 172) R-squared = 0.323 | | |  |  |
| Intercept | -0.08 | 6.54 | -13.16, 12.66 | 5457 |
| N added | -1.06 | 0.55 | -2.13, 0.00 | 23471 |
| P added | 0.19 | 0.55 | -0.88, 1.26 | 21934 |
| **N added : P added** | **-1.33** | **0.67** | **-2.64, -0.01** | **22041** |
| log(GS) | 0.02 | 0.72 | -1.40, 1.44 | 17588 |
| **log(GS) : N** | **1.74** | **0.48** | **0.81, 2.68** | **23141** |
| log(GS) : P | 0.57 | 0.48 | -0.38, 1.52 | 22117 |
| log(GS) : N : P | 0.76 | 0.61 | -0.43, 1.95 | 22723 |
