# Supplementary material for: Genome size influences plant growth and biodiversity responses to nutrient fertilization in diverse grassland communities
Source: PLoS Biol. 2024 Dec 11;22(12):e3002927. doi: 10.1371/journal.pbio.3002927 (PMC11633961; doi:10.1371/journal.pbio.3002927)
Supplement: S4 Table — (a, b) The ANOVA output (a) and summary table (b) for a linear mixed-effects model fitting the effect of N and P fertilization and plot age on the change in cover-weighted GS, compared to control plots on 27 sites in the Nutrient Network (ΔcwGS(control vs. treated), n = 589). Significant differences are shown in bold and starred (* = p ≤ 0.05, ** = p ≤ 0.01, *** = p ≤ 0.001). (c, d) The ANOVA output (c) and summary table (d) for a linear mixed-effects model fitting the effect of N and P fertilization, pretreatment soil N (%), and pretreatment soil P (ppm) on the change in cover-weighted GS, compared to control plots on 20 sites in the Nutrient Network (ΔcwGS(control vs. treated), n = 557). Significant differences are shown in bold and starred (* = p ≤ 0.05, ** = p ≤ 0.01, *** = p ≤ 0.001). (DOCX) [file pbio.3002927.s004.docx]

**S4 Table ANOVA outputs of post-hoc weighted genome size (GS) models**

**a** ANOVA table for the effect of N and P fertilisation and plot age on ΔcwGS_(control vs treated)_.

R^2^ = 0.091.

| **ΔcwGS (control vs treated) ~** | **Sum Sq** | **Mean Sq** | **df** | **F-value** | **p-value** |  |
| --- | --- | --- | --- | --- | --- | --- |
| N added | 0.001 | 0.001 | 1, 587 | 0.02 | 0.899 |  |
| P added | 0.011 | 0.011 | 1, 587 | 0.12 | 0.724 |  |
| Plot Age | <0.001 | <0.001 | 1, 59 | <0.01 | 0.963 |  |
| N added : P added | 0.107 | 0.107 | 1, 589 | 1.19 | 0.276 |  |
| **N added : Plot Age** | **0.569** | **0.569** | **1, 586** | **6.32** | **0.012** | ***** |
| P added : Plot Age | 0.008 | 0.008 | 1, 586 | 0.09 | 0.770 |  |

**b** Summary table for the effect of N and P fertilisation and plot age on ΔcwGS_(control vs treated)_.

| **ΔcwGS (control vs treated) ~** | **Estimate** | **Standard Error** | **df** | **t-value** | **p-value** |  |
| --- | --- | --- | --- | --- | --- | --- |
| No nutrients added | -0.033 | 0.065 | 64 | -0.51 | 0.610 |  |
| N added | 0.008 | 0.063 | 587 | 0.13 | 0.899 |  |
| P added | 0.022 | 0.063 | 587 | 0.35 | 0.725 |  |
| Plot Age | <0.001 | 0.007 | 59 | 0.05 | 0.963 |  |
| N added : P added | 0.050 | 0.046 | 589 | 1.09 | 0.276 |  |
| **N added : Plot Age** | **0.015** | **0.006** | **586** | **2.51** | **0.012** | ***** |
| P added : Plot Age | 0.002 | 0.006 | 586 | 0.29 | 0.770 |  |

**c** ANOVA table for the effect of N and P fertilisation, soil N and soil P on ΔcwGS_(control vs treated)_, . R^2^ = 0.101.

|  |  |  |  |  | |  |  |
| --- | --- | --- | --- | --- | --- | --- | --- |
| **ΔcwGS (control vs treated) ~** | **Sum Sq** | **Mean Sq** | **df** | **F-value** | | **p-value** |  |
| N added | 0.040 | 0.040 | 1, 477 | | 0.38 | 0.032 |  |
| P added | 0.025 | 0.025 | 1, 483 | | 0.24 | 0.402 |  |
| Soil N (%) | 0.020 | 0.020 | 1, 121 | | 0.19 | 0.636 |  |
| Soil P (ppm) | 0.021 | 0.021 | 1, 95 | | 0.20 | 0.639 |  |
| N added : P added | 0.321 | 0.321 | 1, 477 | | 3.09 | 0.058 |  |
| N added : Soil percent N | 0.003 | 0.003 | 1, 478 | | 0.03 | 0.963 |  |
| P added : Soil percent N | 0.282 | 0.282 | 1, 493 | | 2.72 | 0.110 |  |
| **N added : Soil P** | **0.532** | **0.532** | **1, 483** | | **5.13** | **0.025** | ***** |
| P added : Soil P | 0.019 | 0.019 | 1, 482 | | 0.18 | 0.759 |  |
| N : P : Soil percent N | 0.201 | 0.201 | 1, 481 | | 1.94 | 0.162 |  |
| N : P : Soil P | 0.006 | 0.006 | 1, 479 | | 0.06 | 0.732 |  |

**d** Summary table for the effect of N and P fertilisation, soil N and soil P on ΔcwGS_(control vs treated)_, . R^2^ = 0.101.

| **ΔcwGS (control vs treated) ~** | **Estimate** | **Standard Error** | **df** | **t-value** | **p-value** |  |
| --- | --- | --- | --- | --- | --- | --- |
| No nutrients added | -0.078 | 0.064 | 67 | -1.22 | 0.228 |  |
| N added | 0.040 | 0.072 | 477 | 0.62 | 0.535 |  |
| P added | -0.035 | 0.072 | 483 | -0.49 | 0.622 |  |
| Soil N (%) | 0.062 | 0.142 | 122 | 0.44 | 0.664 |  |
| Soil P (ppm) | <0.001 | <0.001 | 95 | 0.45 | 0.658 |  |
| N added : P added | 0.181 | 0.103 | 477 | 1.76 | 0.079 |  |
| N added : Soil percent N | -0.027 | 0.163 | 478 | -0.17 | 0.867 |  |
| P added : Soil percent N | 0.272 | 0.165 | 493 | 1.65 | 0.100 |  |
| **N added : Soil P** | **0.002** | **0.001** | **483** | **2.27** | **0.024** | ***** |
| P added : Soil P | <0.001 | <0.001 | 482 | -0.43 | 0.669 |  |
| N : P : Soil percent N | -0.318 | 0.228 | 481 | -1.39 | 0.164 |  |
| N : P : Soil P | <0.001 | 0.001 | 479 | -0.25 | 0.803 |  |
