# Supplementary material for: Genome size influences plant growth and biodiversity responses to nutrient fertilization in diverse grassland communities
Source: PLoS Biol. 2024 Dec 11;22(12):e3002927. doi: 10.1371/journal.pbio.3002927 (PMC11633961; doi:10.1371/journal.pbio.3002927)
Supplement: S5 Table — Results of a linear model showing the relationship between log-transformed genome size (GS) and cell density across 6 sites in the Nutrient Network (n = 81, sites: Cedar Creek, Chichaqua Bottoms, Kellogg, Konza, Spindletop, and Temple). Significant differences are shown in bold and starred (* = p ≤ 0.05, ** = p ≤ 0.01, *** = p ≤ 0.001). (DOCX) [file pbio.3002927.s005.docx]

**S5 Table: Relationship between cell density and genome size**

F_1,77_ = 12.58, p = <0.001, R-squared: 0.129

| **Stomatal Density ~** | **Estimate** | **Standard Error** | **t-value** | **p-value** |  |  |
| --- | --- | --- | --- | --- | --- | --- |
| **Intercept (low GS)** | **15248.71** | **1803.81** | **8.45** | **<0.001** | ******* | |
| **Log GS** | **-3241.18** | **913.89** | **-3.54** | **<0.001** | ******* | |
