# Supplementary figures and images for: Genome size influences plant growth and biodiversity responses to nutrient fertilization in diverse grassland communities
Source: PLoS Biol. 2024 Dec 11;22(12):e3002927. doi: 10.1371/journal.pbio.3002927 (PMC11633961; doi:10.1371/journal.pbio.3002927)

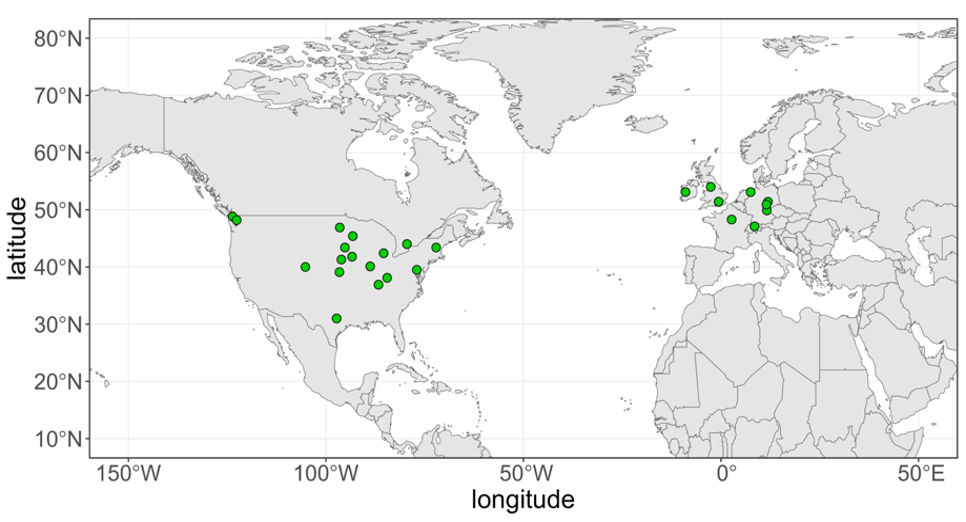

Supplement: S1 Fig — Sites form part of the Nutrient Network, a global collaborative network of experimental fertilized grassland field trials, and are indicated by green circles. The map was produced from a Natural Earth data shapefile (www.naturalearthdata.com) and compiled and plotted using the rnaturalearth and ggplot2 R-packages. (TIFF) [file pbio.3002927.s006.tiff]

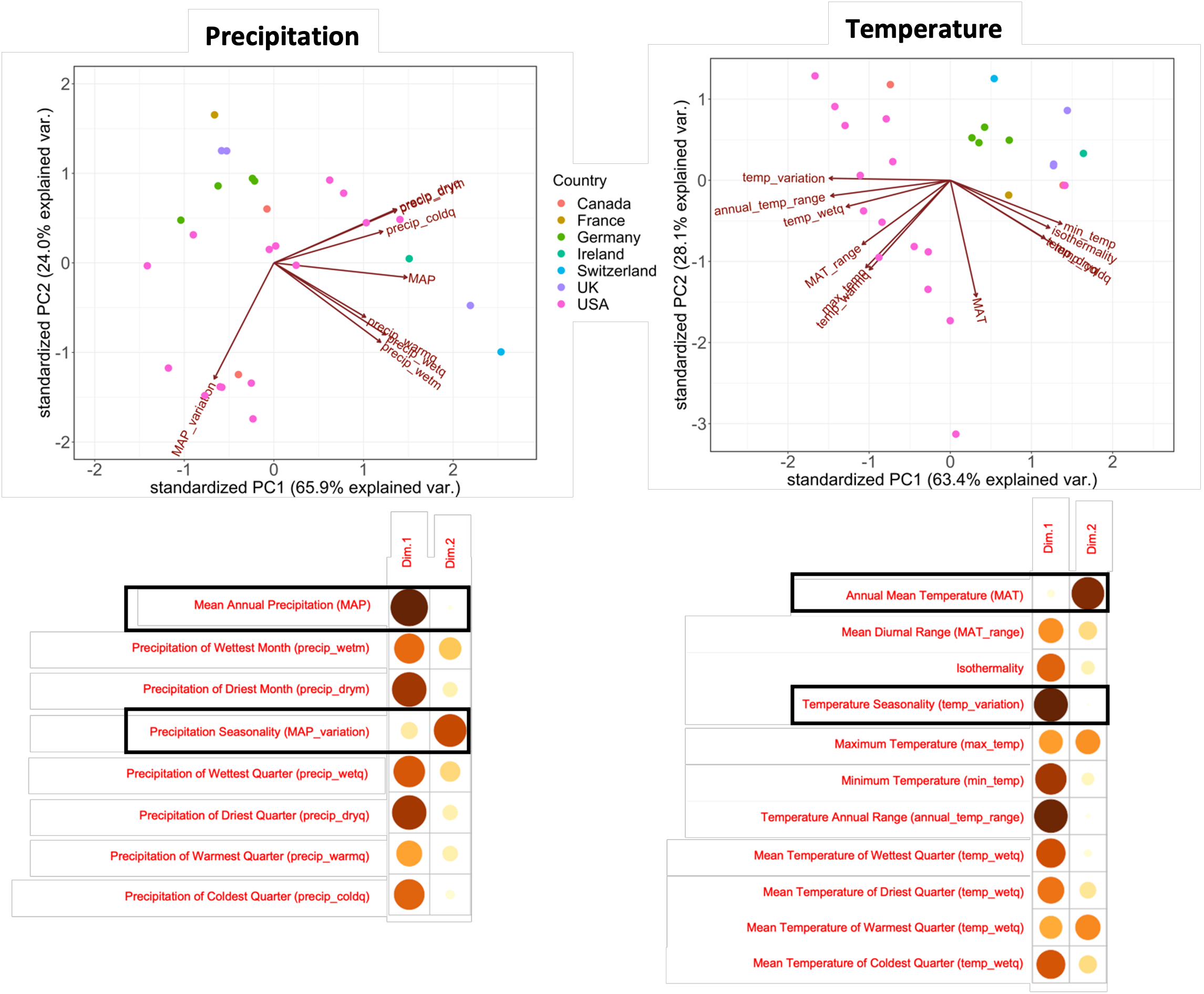

Supplement: S2 Fig — The 20 BioClim variables (displayed above) from WorldClim v.2 were extracted for each site at the 30 arc second scale. Principal component analysis was used to identify variables that explained the largest proportion of variation in precipitation and temperature across the 27 sites studied. PCA plots split into precipitation (left) and temperature variables (right). Coordinates of sites are labeled and are colored by country. The contribution of each variable to the principal components (PC) 1 and 2 are indicated by the direction and length of the arrows. Loadings are shown in the tables, with the size and depth of color of the circles indicating the contribution of each climatic variable to the 2 PCs. Variables that contributed most substantially (boxed variables) in PC1 (Dim.1) and PC2 (Dim.2) were chosen as proxies for the 4 climatic factors—temperature, precipitation, temperature seasonality, and precipitation seasonality. The data underlying this figure can be found at https://doi.org/10.6073/pasta/0d6b08fbcf08605881edfb7acf0a1741. (TIFF) [file pbio.3002927.s007.tiff]

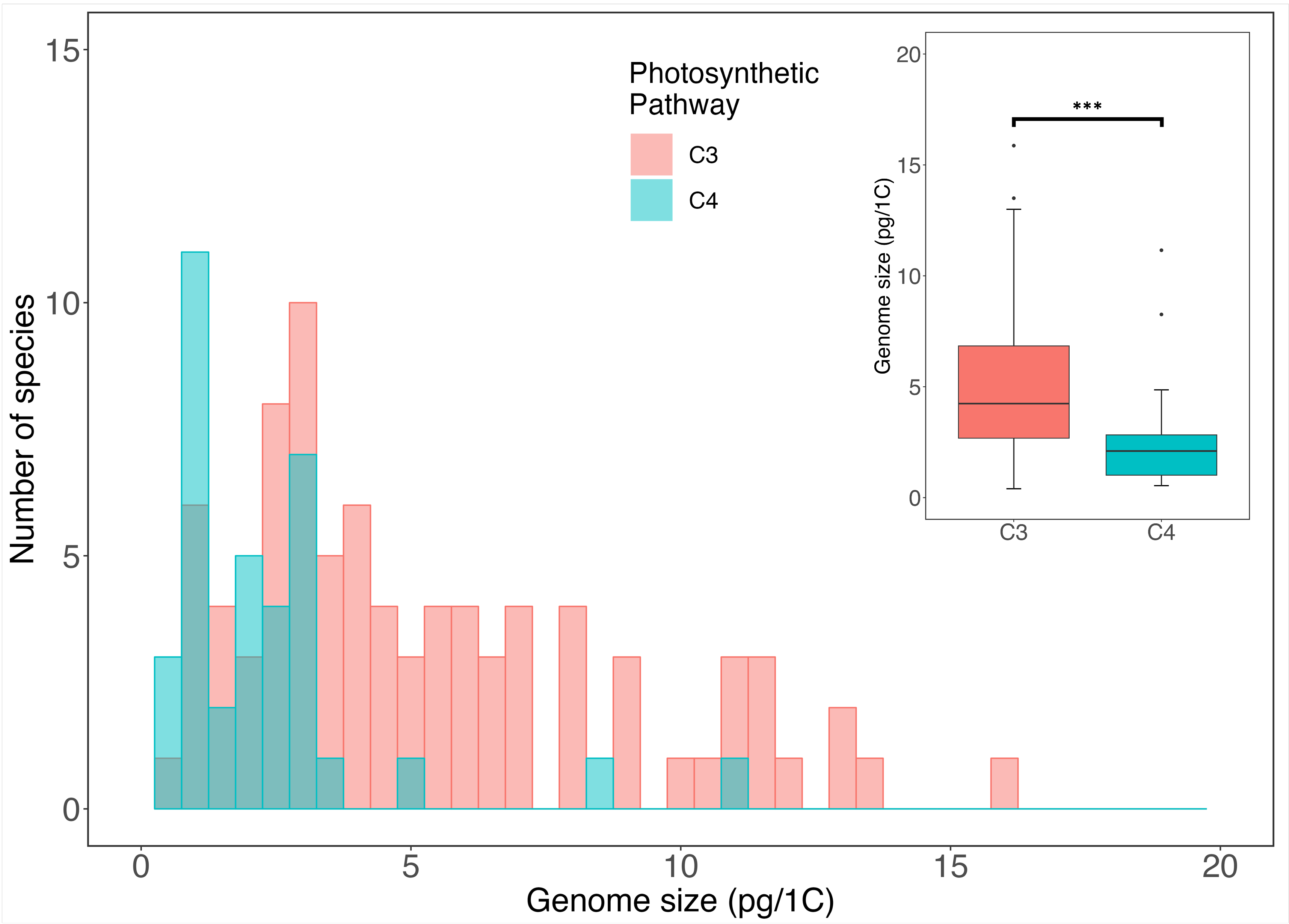

Supplement: S3 Fig — The histogram shows the distribution of genome size (GS) across C3 and C4 grasses, colored by photosynthetic pathway. The inlaid boxplot shows the average and range of GS of grasses for each photosynthetic-type category, with the significant difference indicated by significance stars (p < 0.001). n = 86 (C3), 35 (C4). The data underlying this figure can be found at https://doi.org/10.6073/pasta/0d6b08fbcf08605881edfb7acf0a1741. (TIFF) [file pbio.3002927.s008.tiff]

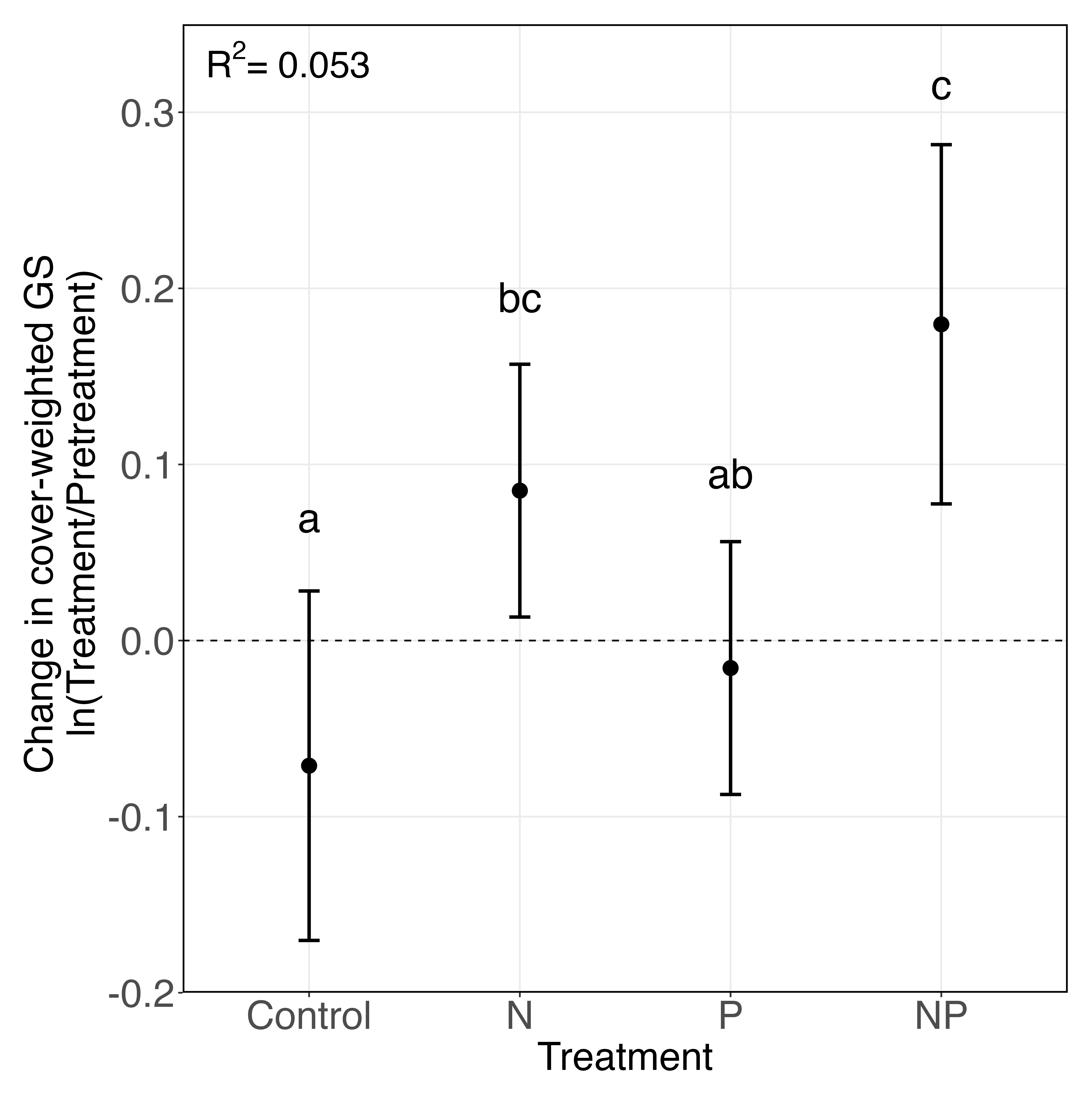

Supplement: S4 Fig — Average cover-weighted genome size (cwGS) was calculated for plots under factorial N and P treatment, using a 3-year mean for species percentage cover. Log response ratios (LRR) of cwGS relative to pretreatment were calculated to measure temporal changes in genome size (GS) in response to fertilization. Error bars indicate 95% confidence intervals. Significant differences between treatments are indicated by letters (Tukey’s HSD test p < 0.05) and the R2 value for the fitted linear mixed-effects model fitted for this data is displayed (n = 597). The data underlying this figure can be found at https://doi.org/10.6073/pasta/0d6b08fbcf08605881edfb7acf0a1741. (TIFF) [file pbio.3002927.s009.tiff]

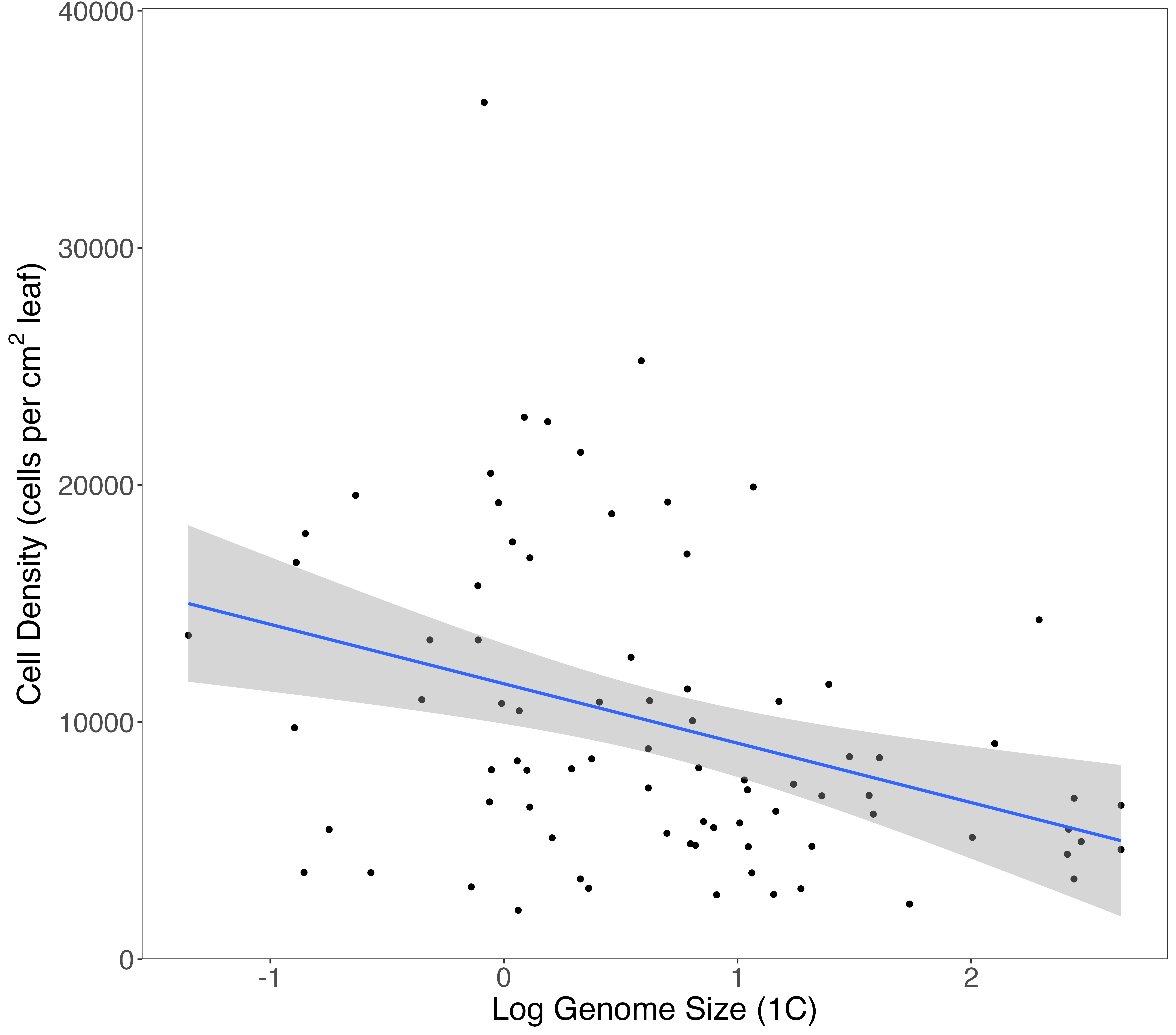

Supplement: S5 Fig — Cell density (per cm2 fresh tissue) was compared between 63 species of varying genome size across 6 sites in the Nutrient Network (Cedar Creek, Chichaqua Bottoms, Kellogg, Konza, Spindletop, and Temple). The solid blue line indicates the significant negative relationship, with the gray region representing 95% confidence intervals. The data underlying this figure can be found at https://doi.org/10.6073/pasta/0d6b08fbcf08605881edfb7acf0a1741. (TIFF) [file pbio.3002927.s010.tiff]
