# Supplementary material for: Genome size influences plant growth and biodiversity responses to nutrient fertilization in diverse grassland communities
Source: PLoS Biol. 2024 Dec 11;22(12):e3002927. doi: 10.1371/journal.pbio.3002927 (PMC11633961; doi:10.1371/journal.pbio.3002927)
Supplement: S1 Data — A more detailed description of the methods, standards, and buffers used in measuring plant GS. (DOCX) [file pbio.3002927.s011.docx]

**S1 Data: GS methods, standards and buffers**

GS values (expressed as pg/1C) were obtained using a one-step flow cytometry procedure [1]. Approximately 1 cm^2^ of leaf material from both the sample and an internal calibration standard were chopped in isolation buffer, with both the buffer and standard used being determined empirically for each species and material type. The suspension was then filtered through a 30 μm nylon mesh (Sysmex, Goritz, Germany) and nuclei were stained with propidium iodide. The relative fluorescence of 2000-3000 particles was recorded using a flow cytometer (Sysmex CyFlow Space Partec GmbH, Germany for samples from Europe and Accuri Inc, Ann Arbor, Michigan, USA for samples from North America). Fluorescence peaks were visualised as histograms in machine-specific software and, after using gating to remove debris, mean counts and coefficient of variations (CV) were derived from these histogram plots. All low-quality samples that had peak CVs of of more than 5% were re-run. The following formula was used to convert the relative fluorescence values into 1C DNA content (in pg).

$$Sample 1C DNA value:= \frac{Standard 2C DNA value*\left( \frac{Sample 2C mean peak position}{Standard 2C mean peak position} \right)}{2}$$

GS measurements and CV values, as well as GS sources, broken down by species and site, are in the supplementary xlsx file. The buffers and standards used and their abbreviations are as described below.

The buffers used are as follows:

GPB = General Purpose Buffer [2] supplemented with 3% PVP-40 and ß-mercaptoethanol

LB01 = LB01 Buffer [3]

NIB = Modified de Laat’s buffer [4], substituting 15mM

ß-mercaptoethanol for DTT ,supplemented with 0.25mM PVP-40

Solidago = Solidago buffer [5]

WPB = Woody Plant Buffer [2]

The standards used are abbreviated as follows (for GS measurements of standards see [6]):

Glycine *= Glycine max* Merr. “Polanka” (1.25 pg/1C)

Petroselinum *= Petroselinum crispum* (Mill.) Nyman ex A.W.Hill “Champion Moss Curled” (2.25 pg/1C)

Pisum = *Pisum sativum* L. “Ctirad” (4.54 pg/1C)

Raphanus = *Raphanus sativus* L*.* “Saxa” (0.55 pg/1C)

Solanum  *= Solanum lycopersicum* L. “Stupické polní rané” (0.98 pg/1C)

Vicia *= Vicia faba* L. “Inovec” (13.45 pg/1C)

Zea  *= Zea mays* L. *“*CE-777” (2.71 pg/1C)

Supplementary Data references:

1. Doležel J, Greilhuber J, Suda J. Estimation of nuclear DNA content in plants using flow cytometry. Nat Protoc. 2007;2: 2233–2244. doi:10.1038/nprot.2007.310

2. Loureiro J, Rodriguez E, Doležel J, Santos C. Two new nuclear isolation buffers for plant DNA flow cytometry: a test with 37 species. Ann Bot. 2007;100: 875–88. doi:10.1093/aob/mcm152

3. Doležel J, Binarová P, Lucretti S. Analysis of Nuclear DNA content in plant cells by Flow cytometry. Biol Plant. 1989;31: 113–120. doi:10.1007/BF02907241

4. de Laat AMM, Blaas J. Flow-cytometric characterization and sorting of plant chromosomes. Theoretical and Applied Genetics. 1984;67: 463–467. doi:10.1007/BF00263414

5. Bharathan G, Lambert G, Galbraith DW. Nuclear DNA content of monocotyledons and related taxa. Am J Bot. 1994;81: 381–386. doi:10.1002/j.1537-2197.1994.tb15457.x

6. Pellicer J, Leitch IJ. The application of flow cytometry for estimating genome size and ploidy level in plants. Besse P, editor. Methods in Molecular Biology. 2014;1115: 279–307. doi:10.1007/978-1-62703-767-9_14
