# Supplementary material for: Genome size influences plant growth and biodiversity responses to nutrient fertilization in diverse grassland communities
Source: PLoS Biol. 2024 Dec 11;22(12):e3002927. doi: 10.1371/journal.pbio.3002927 (PMC11633961; doi:10.1371/journal.pbio.3002927)
Supplement: Attachment — Submitted filename: Response to Reviewers.docx [file pbio.3002927.s013.docx]

We thank you and the referees for the careful examination and critique of our manuscript. We have addressed the comments below and in the manuscript. The referees’ comments are in blue and our responses are in black. Any line references by the authors refer to the line numbers in the revised manuscript, with changes highlighted.

**Reviewer #1:**

The manuscript uses a northern hemisphere grassland nutrient addition experiment to test hypotheses that suggest plants with larger genomes will be more responsive (larger) following N/P enrichment in certain environments, compared with small genome taxa, based on earlier studies at single sites and using simple experiments. The strengths of the study are the use of data from multiple natural grasslands, the range of functional groups involved, incorporation of several climate parameters, and the use of plant response data collected over several years. Overall, I have no problems with the study, data analysis and interpretation. It's a useful addition to studies defining the ecological niche of taxa with large genomes, often polyploids, in natural ecosystems. I support its publication.

We thank the reviewer for these supportive comments

I offer the following general comments for consideration by the authors during revision.
1 The plant response measure relies on estimates of cover which are very difficult for grasses and often ignore height gain. This may advantage species with lateral spread and larger leaves, biasing some groups. Could this be relevant?

While we feel that cover can give a better indication of the dominance structure on plots, we acknowledge that the advantage to laterally spreading plants should be a consideration when using cover as a measure of plant growth response. We have included a discussion of this in the Discussion lines 269-272.

2 I could not find any data on the soil nutrient status at the sites (N/P) which could explain some of the noise in the response data. Is this not available?

We agree that soil nutrient status does explain some of the noise in the response data, and consider this is a useful addition to the manuscript. Pretreatment soil data was available for 20 of the 27 sites, so we have now included the results of a linear mixed effect model fitting the effect of N addition, P addition and pretreatment N and P on the change in cover-weighted genome size. This is included in supplementary Table S4, the Methods (lines 476-479) and in the Discussion in the context of understanding the more prominent effect of N rather than P fertilisation (lines 260-262).

3 Check throughout for consistent use of plant response terms: growth, performance, fitness etc. to ensure these are accurate.

We thank the reviewer for this and have gone through the manuscript to ensure such terms are used consistently.

4 In the cover weighted genome size determinations, were the same taxa involved in each treatment/control over time? Did the controls have a time aggregation similar to the other treatments when determining the cover weighted genome sizes? In other words did nutrient addition facilitate new incursions of large genome taxa, or just facilitate extra growth of established species?

In the weighted genome size determinations, the average was taken for all species sampled across all three years, even when some species appeared and disappeared (i.e. cover = 0) between years.

From the data, differences in cover for large GS species were driven by both the extra growth of established species and by the recruitment of species with large genomes from outside the plot (including those from elsewhere on the site), although extra growth of established species dominated the datasets. No compositional analyses were done, but these potential explanations for differences in weighted genome size have been highlighted in the Results with examples (see lines 148-152).

For the control vs treated measures of cover-weighted genome size, the controls had a time aggregation similar to the other treatments (i.e. both control and treatment values were three year means), as all plots were run simultaneously over the years of study. We have clarified this in the Methods (lines 401, 453-455).

5 Do you think the non-significant response of annual forb, geophyte etc merely reflects their low occurrence values in the plots?

Their low occurrence in the plots may contribute to the responses we observed. While we previously mentioned this in the Discussion, we have now expanded upon it (lines 341-345).

6 Line 304. Power issue is really "initial advantage" or a "priority effect".

We feel that the power issue is appropriate in this instance, as we discuss the impact of the number of species, sites and the variation in GS on the ability to detect a significant difference where patterns emerge. We have edited it to now read “statistical power issue” (line 334).

7 Do the authors have any explanation from their results for why natural grasslands in their study maintain a combination of both small and large genome species in N deficient soils? This suggests that large genome species can survive and reproduce at low N/P (i.e. have reasonable fitness), even if they do not achieve maximum growth.

We agree with the reviewer that species with larger genomes are able to survive and grow in N deficient soils, but we posit that their increased nutrient demands might constrain their growth rates under such conditions, and/or prevent them from being as competitive as they could be, influencing competitive dynamics. This means that they are less dominant than those with smaller genomes in N-deficient grassland communities. We have made this point clearer in both the Introduction (lines 48-51) and Discussion (lines 232-234).

**Reviewer #2:**

With 27 grassland sites from the NUTNET work, this study reported that N fertilization stimulated the growth of species with larger genome size, a finding has been reported by many local experiments. The new result from this study is that the interaction between GS and N response would be climate-dependent and plant group-dependent.

1 This manuscript wants to examine the differences of larger GS plants and smaller GS plants in their responses to fertilization, and have talked much about large and small GS. Unfortunately, I did not find the definition or standard to classify different species into small and large GS plants. Such standard is the basis for the credibility of findings from this study.

Since genome sizes fall along a broad spectrum, we feel that classifying species into “large” or “small” GS categories may not be helpful, as GS variation within the “small” or “large” could still be sufficient to generate nutrient-GS interactions. Indeed, we could then be criticised for choosing arbitrary thresholds. The focus of this study is on the differential responses of species to nutrient addition based on their genome size **relative** to other species on the same site i.e. those that they are competing with. The variation in average genome size between sites also means that a species that may be considered to have a relatively small GS compared to other species on one site may have a relatively large GS compared to other species on another site. Furthermore, because GS variation arises as a consequence of evolutionary processes, we controlled for phylogenetic signal when analysing the GS continuum that occurs. We acknowledge that this was not clearly explained, and we have altered language throughout to make it clearer we are talking about comparatively larger/smaller genome sizes, rather than arbitrarily defined “large” or “small” GS (e.g. lines 23-31, 48-53).

2 Authors reported the GS values for 597 species (right?), and only GS data for 469 species were used in further analyses. Given that this study focused on the role GS in regulating plant growth in response to nutrient addition, it would be better to keep consistent for the data. If the 597 GS values are for the 469 species, it is important to clearly state how to assign more than one values to a particular species.

Where possible we have endeavoured to use site-specific genome size values for species, using samples collected from 12 of the 27 sites (see Methods). As some species were sampled on multiple sites we therefore have multiple GS values for some species, with each site sampled having its own GS value for that species resulting in the 597 specific GS values representing 469 different species. We have clarified this in the Results (lines 122-125) and Methods (lines 390-395). The origin and use of each GS value is also given in the Supplementary Data file ‘GS methods, standards and buffers’.

3 Authors classified all the plants into different groups, including grass, annual forb, perennial forb, legume. Is there any overlap for species in Geophyte and other group, such as grass, perennial forb, and legume? I don't think the Geophyte group is from the same classification system with grass, forb, and woody species.

We agree with the reviewer that some species could be classified into multiple categories. However, in this instance we assigned only one functional group to each species, using the functional group classification system used by the Nutrient Network, to assure consistency across the 27 sites. This has been clarified in the Methods (lines 406-407).

4 The dependence of the impacts of N fertilization on cwGS on precipitation and temperature seasonality is one of the important findings from this study. Thus, a figure is needed to show the results clearly, rather than showing the interactive results in Table 1.

We agree that precipitation and temperature seasonality are important findings of our study and agree that a figure would make these results clearer. We have therefore included a new figure (Fig. 4) with four graphs accompanying the results shown in Table 1. Four separate graphs were used as the effects of each climate variable on nutrient-GS interactions were examined separately.

5 Similarly, for the result of significant interaction between log:N in Table 2. While the results showed a significant interaction between Log GS and N fertilization, that does not mean species with larger GS would show greater increase in response to N fertilization, as stated in Lines 174-176. Figure showing such a pattern is needed.

The “estimate” given In Table 2 gives the effect size, meaning it does indicate the direction of the response to fertilisation. The log(GS) : N interaction estimate given in the table shows that increases in genome size are correlated with an increase in percent cover on plots treated with N (both N and N and P). Specifically, the model predicts that for every increase in log(GS) of 1, a species will increase in percent cover by an additional 1.27% in response to N addition (with some uncertainty indicated by the 95% Credible Intervals).

We feel a figure for these data adds little beyond what is apparent in the table. Nevertheless, we attach an example of a potential figure below for reference (solid lines indicate relationships with 95% credible intervals that do not overlap 0) should you or the referee think it is necessary to include in the manuscript or as a Supplementary Figure.

6 The same problems are for the results in lines 185-193. The statistic results just showed the significance, but did not show any pattern for the changes described here.

We refer the reviewer to the response to the comment above.

7 Three hypotheses were proposed in the Introduction, but were not revisited in the Discussion.

We agree with the reviewer that tying the hypotheses through the paper improves its clarity, and have revisited them more clearly in the Discussion (lines 205-209, 276-279, 307-309), as well as structuring each of the main Discussion headings around the 3 hypotheses.

8 Title: 'plant growth' would be clearer than 'growth'

We agree and have amended the title.

9 Lines 12-14 How about fertilization with other elements? Authors ascribed the positive response of larger genome plants to the release from nutrient limitation after nitrogen fertilization. Did nitrogen fertilization increase the availability of nutrients beyond nitrogen?

Our core hypotheses focussed on the effect of nitrogen and phosphorus as these are the most prominent macronutrients associated with nucleic acid production, and are elements which increase at the cellular level with increasing genome size, Our study builds on past work that studied these macronutrients [1,2]. We agree that the question of whether an increased availability of other elements beyond phosphorus is facilitated by nitrogen addition deserves further exploration, though such an analysis was not conducted in this work.

10 Line 28-33 Authors cited many individual studies from different sites. Are all the studies following the same standard for classifying smaller and larger genome size plants?

Some studies have used classifications of “large” and “small” genomes for parts of their analysis [1] and others have been comparing individual cytotypes of a species [3] reflecting the ranges of GS on the single site plots being studied. But here the ranges differ substantially between sites, and so we focussed on whether species have smaller or larger GS relative to other species with which they coexist and potentially compete, as discussed in relation to the comment above. We think this is more meaningful for considering the impact of GS on how plants respond to nutrient additions and plant community dynamics.

11 Line 42 NUTNET has investigated more beyond biodiversity.]

We have amended the line (now line 102-106) to better encapsulate the key goals of the Nutrient Network

12 Line 68 delete the comma

Thank you, the comma has been removed.

13 Line 78 I am afraid that you are talking about intra-annual fluctuations.

Thank you, this has been amended (now line 75)

14 Line 92 Under which condition would small GS annuals might be advantageous?

This point has been expanded upon (lines 89-92) to highlight this.

15 Line 94 Why species with higher N-use efficiency respond less to P fertilization?

Thank you, this was a typo that has been corrected.

16 Line 143 It is not safe to say NP plots had higher GS than N plots, as there is no statistical difference as shown in Fig 3.

We agree that the wording here was unclear, and have therefore modified the language to highlight that this is more speculative (line 152-154).

17 Line 197 'series' or 'species'? I think you used 'species' in the Abstract.

In this situation, “diploid-polyploid comparisons” may better explain the experiment, we have thus amended the Abstract accordingly (line 2), as well as elsewhere.

18 Line 243 But the work of Fay et al (2015 Nature Plants), also based on NUTNET sites, showed multiple nutrient limitation is widespread in the NUTNET grasslands.

This has been acknowledged in the discussion (lines 255-257).

19 Line 314-315 did P addition alter plant community based on GS?

We have altered the wording from “nutrient availability” to “nitrogen availability” to better reflect the findings of this study (line 349)

**Reviewer #3:**

The paper examines response of species of different nuclear genome sizes to nitrogen and phophorus addition in grassland communities, based on a large scale fertilization experiment. The whole study is sound, although some of the assumptions/hypotheses made in the introduction are questionable and may need better support (see below). I have a number of comments to the methods section, but most of them can be handled by better explanation/description of the data handling/analysis procedures.

Thank you for your feedback.

1 I would also appreciate discussion of the absence of the effect of phosphorus addition - one would expect that P-limitation may be as important as the N-limitation (given the high amount of P in DNA). Were all the sites only nitrogen limited? One would appreciate seeing also simple response in species composition to nitrogen and phosphorus addition as a background information.

We had expected to see N and P colimitation, although past work on an Inner Mongolia grassland site demonstrated a predominant effect of N and a smaller addition effect of N and P together [4]. There is some indication of N and P colimitation and we have now incorporated some discussion on the absence of an effect of P addition, bringing into context too some additional analysis that incorporated soil N and P data, which suggest the weak N:P effects (lines 250-263).

Specific comments:

2 li 31: there references are papers dealing only with ploidy effects, not fully relevant to support the argument

We have now clarified that they are comparing diploids and polyploids in the text (line 28-29).

3 li 52: this is true for water and nutrients, but is less true for light due to strong asymmetry in competition for light. See e.g. de Malach et al. Jecol 2016.

Thank you for highlighting this. We have altered the wording in this paragraph (line 44-45) and have now included this useful paper within the bibliography.

4 li 58: it is true that larger-GS species may initially grow faster due to the potential of (large) cell expansion, but growth by cell expansion is limited and cannot be maintained for long as the cell volume cannot grow indefinitely. Further size increase must be attained by cell division which takes longer in large GS species and is likely to counteract the positive effect of cell expansion. One may therefore expect that the relationship may be nonlinear or even unimodal. It be worth to explore potential nonlinearity here.

We agree of course that cell expansion cannot be the sole mechanism for growth, and that cell expansion-cell division trade-offs may generate some non-linearity in the results. However, we argue that this initial growth by cell expansion may in itself be sufficient to generate a competitive advantage that gets maintained by the shading out of competitors – this is also found and discussed in Peng *et al.* [4] who measured the amount of light passing through the canopy in their grassland nutrient experiment.

5 Actually, I would appreciate more info on distribution of GS in the main text (histograms, etc.), so that the reader can appreciate the range of GS values covered by the species involved in the study.

Details on the distribution of GS are given in the results (lines 125-129) and histograms both across all species, and within each functional group, are now given as Figure S3.

6 li 89: is there a good evidence that these organs store also soil-borne nutrients in addition to the stored carbon?

Relevant citations have been included in the text, showing (i) the allocation of nutrients to storage organs and (ii) the use of storage organs as a temporary store of nutrients before reallocation to other organs within the plant (line 94).

7 li 261: but larger cells would mean also larger stomata and potential higher water loss

Larger stomata do not necessarily have higher water loss as any increased transpiration is overcome by a lower stomatal density. In the introduction we have some text showing that the effects of GS on water-use efficiency remains unclear. We provide some new cell density data to accompany the text in the Discussion, and make it clear that cell density is inversely proportional to cell size (Fig. S5 lines 241-244, see also reviewer 4, point 1). Also, unpublished data suggests that transpiration is more heavily influenced by Mean Annual Precipitation than stomatal characteristics.

8 li 326 how many sites were included in the study? There is no info in the methods; the discussion says 27, but the residual d.f. in the Table 1 (for climatic variables, which are site-defined) seem to indicate larger values.

27 sites were included in the study (this has been added into the Methods at line 362). The difference in df values are due to the model being a mixed-effects model, including block as a random factor.

9 li 337: how representative is the Kew Plant DNA database? One can imagine that if plant of either small or large genome size are preferntially measured, this procedure may introduce bias into the data.

The Kew Plant DNA C-values database represents the broadest compilation of GS data across angiosperms currently available, comprising over 12,000 species. Since the GS of a species is typically unknown before a undertaking a GS analysis, there is limited scope, or no scope for ascertainment bias. Only GS values for species found at Nutrient Network sites were used. We agree with the reviewer that, where cytotype variation exists, the choice of larger or smaller GS values could bias the data. We have therefore outlined in the Methods (lines 377-382) that for the 33 species where multiple values were present, that the smaller (diploid) value was consistently used. This is the most conservative approach for this analysis (see also point 11, below). The potential consequences of this methodology are raised in the Discussion (lines 222-227). We also included analysis for 12 sites from which we used only directly measured, site-specific values (**Table S2F-I**), for which we found comparable, if not stronger results that are in line with what the core analysis is demonstrating (see lines 227-231).

10 li 342: were these multiple values indicative (in all cases) of polyploid series?

They were indicative in all cases, and we have strengthened the language accordingly (line 378).

11 li 343: "the most conservative estimate" - why the small GS size should be considered conservative? It this may introduce potential bias by falsely assigning species to diploid status

We agree that assigning cytotype status can introduce potential biases. However, we consider our approach is being ‘conservative’, as assigning larger GS cytotypes to a taxon, in the absence of other information, may result in the effect of GS on nutrient response being over-estimated, while by choosing smaller GS it will likely be under-estimated, as discussed in the Methods (lines 377-382) and Discussion (lines 222-231). By consistently choosing the smaller GS value we aim to control the manner in which potential biases may be introduced into the analysis. As said above, we also included analysis for 12 sites from which we used site-specific values, and came to the same or stronger results, reinforcing our inference that our approach is ‘conservative’.

12 li 347: how were these species selected? To attain a target proportion at each site or plot? Or taking into account also their presence in individual treatments?

The species used for site-specific genome size measurements were those sampled on 12 sites by site coordinators and co-authors during the time period of data collection (2020-2022). These sites were chosen as both those that had lower proportions of species with available GS data and to enable as many sites as possible to be sampled. We have altered some wording in the Methods to help clarify how species were selected (lines 384).

13 li 361: it would be good to know how many years have elapsed between these three most recent years and the start of the treatment (i.e. for how long time the treatment has been applied).

The treatment duration (given as first and last year of analysis) has now been included in Table S1, and some *post hoc* analysis has now been included in the Discussion (lines 227-231), showing that the effects of N on cover-weighted GS is strengthened in older plots compared to younger plots (see **Table S4C-D**).

14 li 385: phylogenetic signal was calculated in log-transformed GS or not?

The phylogenetic signal was taken on non-log transformed values. This has been clarified in the text (lines 427-429).

15 li 389: the cover-weighted GS was log-transformed before calculation? If not, the high positive skew in the GS may highly distort the outcome.

The GS data was log-transformed prior to the calculation of cwGS. This has now been included in the Methods (lines 457-459) – thank you for spotting that was missing!

16 li 397: why this has not been detemrined using phylogenetic generalized least squares? Given the high phylosignal in GS it should be appropriate.

We have now used pgls to analyse these data, giving the same result. The updated methodology and statistics have been added to the Methods (lines 443-444) and Results (lines 130-132).

17 li 403: this is unclear - which patterns were in need of clarification and what clarification means in this context?

We agree this wording was unclear and it has been modified (lines 446-449)

18 409-410. This is unclear

We have reworded and expanded upon this section, we hope it is now clearer (lines 455-459)

**Reviewer #4:**

1 Overall, this is a very interesting dataset that can be used to further our understanding of grassland responses to fertilization.  That said, I disagree with the interpretation of the data.  A major focus of the interpretation of results revolves around the potential impact of genome size on cell size and the subsequent effects of variation in cell size on whole plant physiology and growth. Given that there is no cell size data presented all discussion around cell size as playing a role is purely speculative. I appreciate that the authors cite previous work showing relationships between cell size and genome size but this previous work also shows wide variation in cell size for a given genome size. Without cell size data to back up the inferences made it is hard to accept the author's interpretation of the data.

Thank you for your feedback.

We agree that cell data would strengthen our interpretation of the data and conclusions made. We have therefore now included data on the relationship between cell density (which is inversely correlated with cell size) and GS for 63 species at 6 of our sites studied. We have included a figure showing the negative relationship between GS and cell density in Figure S5, updated the methods (lines 496-503) and discuss this in relation to our data in the Discussion (lines 241-244, 286-287).

2 Further, the analysis presented in the supplementary files seems to suggest that the physiological differences between C3 and C4 grasses may represent an overarching control over the observed GS response, not GS per se. In other words, GS is going along for the ride. Additionally, we know from a long history of C4 research that C4 grasses possess larger mesophyll cells than C3 grasses. What does this mean for arguments made throughout the paper that larger cells lead to advantages in growth (for example lines 57 - 58)?  Should we not expect a greater positive response by C4 grasses than C3 grasses?

We acknowledge that differences between C3 and C4 grasses will influence plants’ responses to nutrients independent of GS. However, an interaction between GS and N addition on percent cover was observed across C3 grasses alone (lines 194-199) indicating the relationship is not solely driven by differences in photosynthetic pathway (**Table S3B**).

Detailed comments below.

3 Figure 1. I did not find this figure very useful for outlining the 3 hypotheses presented in this paper. I do not have specific comments on how to improve the cartoon but my initial interpretation of Fig 1 a story of nutrient addition leading to bigger plants with big genomes that shade out smaller plants with small genomes.

Thank you for your feedback. Based on this, and the inclusion of other data figures within the revised manuscript, this figure has now been removed.

4 Figure 2A. I like seeing the genome size distribution across functional groups, but it is unclear to me why these specific functional groups were chosen.  For example, a distinction is made between annual and perennial forbs but not between annual and perennial grasses, Why? Also, I looked through the data and there are both annual and perennial grasses found in these grassland communities. Additionally, a great deal of text is devoted to discussing differences between C3 and C4 grasses but there is not data presented in Fig 2A for these two functional groups.

We have chosen to separate functional groups based on the categories used by the Nutrient Network (this has now been clarified on line 405-407) and published previously [5,6] to maintain consistency and this included separation of annual and perennial forbs but not annual and perennial grasses. However, we agree that more representation of the C3 and C4 data is warranted given the importance of this factor in our analysis, and have modified Figure 2A (now Figure 1A) to separate the C3 and C4 grasses.

5 Side note - looking through the data I think Sporobolus cryptandrus is C4 and not C3, please double check this.

Thank you for spotting this error. On checking the Osborne dataset it was mislabelled in our dataset and has now been corrected. However, *S. cryptandrus* was one of the species on our NutNet sites for which we were unable to obtain GS data, so it was not included in the analyses.

6 Fig 2B. As stated above it would be good to have a better handle on the representation of C3, C4, annual, and perennial grasses in these systems. Why are these functional groups not represented in Fig 2B? The authors emphasize that "GS-dependent growth responses to nutrients could also vary among plant groups" and spend significant time outlining that C3 and C4 are important functional distinctions to consider. The introduction even cites previous work suggesting that C3 and C4 plants respond differently to N and P fertilization.  Since the authors acknowledge that physiological differences between C3 and C4 plants are important, why is this functional distinction missing from Fig. 2B and instead only highlighted in the supplementary data?

As discussed above, we agree that distinguishing C3 and C4 grasses is important, and thus have modified figure 2B (now Figure 1B) to include distinctions between C3 and C4 grasses.

7 Fig 3. These are very small changes in cover-weighted GS.

We acknowledge that the differences are small but highlight that this is an average observed across 27 complex and very different grassland systems (community composition, climate etc.), where multiple factors will be influencing the growth of plants within these plots. Comments on the magnitude of the change, and some sources of the noise in the data are given in the Discussion (lines 216-231).

8 Lines 204 - 208, 227 - 233:  It would be good to have the nutrient and cell size data for these species when making these arguments.  Without cell size and nutrient data this is all speculative.

As mentioned above, cell density data have been provided and are discussed (lines 241-244, 286-287).

9 Lines 258 - 259:  Are these sites associated with a higher abundance of C4 plants in the control / pretreatment plots?

We agree that the increased abundance of C4 plants in drier grasslands could be a potential explanation for these results, but this does not seem to be the case here. C4 plants were found on 14 of the 27 sites analysed here, including sites with the maximum and minimum values for both precipitation and temperature seasonality observed in the dataset. No significant relationship was observed between mean annual precipitation and the total pre-treatment abundance of C4 plants on a plot, nor for temperature seasonality (see the ANOVA table for linear model below).

| **Total cover of C4 grasses on a plot before treatment~** | **Sum Sq** | **Mean Sq** | **df** | **F-value** | **p-value** |  |
| --- | --- | --- | --- | --- | --- | --- |
| Temperature Seasonality | 2.60 | 2.60 | 1, 11 | 0.007 | 0.937 |  |
| Mean Annual Precipitation | 122.91 | 122.91 | 1, 11 | 0.313 | 0.587 |  |

10 Lines 260 - 262: Again without cell size data this is speculative.

See comments above

11 Line 282 - I think this should be Table S3A and S3B respectively.

This has now been corrected as the table numbers and numbering has changed.

12 Lines 307 - 309:  What does far fewer C4 grasses mean? Are there fewer species, smaller percent cover?  As stated previously please show this data.  This is necessary to better understand how the change in C4 cover influences the observed cover-weighted genome size response to nutrients shown in Fig 3.

The data are now shown in Figure 1A and 1B, and this section has been reworded to clarify the meaning, including changing “far fewer C_4_ grasses” to “fewer C_4_ grass species, occupying only 14 of the 27 sites” (lines 337-340).

References

1.	Guignard MS, Nichols RA, Knell RJ, Macdonald A, Romila CA, Trimmer M, et al. Genome size and ploidy influence angiosperm species’ biomass under nitrogen and phosphorus limitation. New Phytologist. 2016;210: 1195–1206. doi:10.1111/nph.13881

2.	Šmarda P, Hejcman M, Březinová A, Horová L, Steigerová H, Zedek F, et al. Effect of phosphorus availability on the selection of species with different ploidy levels and genome sizes in a long-term grassland fertilization experiment. New Phytologist. 2013;200: 911–921. doi:10.1111/nph.12399

3.	Walczyk AM, Hersch-Green EI. Impacts of soil nitrogen and phosphorus levels on cytotype performance of the circumboreal herb *Chamerion angustifolium*: implications for polyploid establishment. Am J Bot. 2019;106: 906–921. doi:10.1002/ajb2.1321

4.	Peng Y, Yang J, Leitch IJ, Guignard MS, Seabloom EW, Cao D, et al. Plant genome size modulates grassland community responses to multi‐nutrient additions. New Phytologist. 2022;236: 2091–2102. doi:10.1111/nph.18496

5.	Wilcots ME, Harpole WS, Seabloom EW, Borer ET. Community change can buffer chronic nitrogen impacts, but multiple nutrients tip the scale. Ecology. 2021;102: e03355. doi:10.1002/ECY.3355

6.	Garbowski M, Boughton E, Ebeling A, Fay P, Hautier Y, Holz H, et al. Nutrient enrichment alters seasonal β-diversity in global grasslands. Journal of Ecology. 2023;111: 2134–2145. doi:10.1111/1365-2745.14182
